# Supplementary material for: Are carbon emissions trading and green financial instruments synergistic? -Comprehensive quantitative research based on content analysis
Source: PLoS One. 2024 Mar 7;19(3):e0298601. doi: 10.1371/journal.pone.0298601 (PMC10919601; doi:10.1371/journal.pone.0298601)
Supplement: S1 Data — (ZIP) [file pone.0298601.s001.zip › Data/地级市Z_F工作报告.zip!/╜¡╬≈/╕╙╓▌╩╨/└┤╫╘╣1⁄2╓┌║┼í╛┬φ┐╦╩2╛▌═°í┐_2004.docx]

各位代表：

　　本届政府１９９９年６月就职，任期即将届满。现在，我代表赣州市人民政府向市第二届人民代表大会第一次会议报告过去五年的工作，对今后五年的工作提出建议，请予审议，并请市政协各位委员提出意见。

　　一、过去五年的工作回顾

　　五年来，市人民政府在中共赣州市委的领导下，在市人大、市政协的监督和支持下，团结和带领全市人民，解放思想，实事求是，与时俱进，开拓创新，较好地完成了市一届人大各次会议确定的主要任务，开创了我市改革开放和现代化建设的新局面。

　　——过去的五年，是国民经济快速增长、综合实力大为提升的五年。五年中，全市生产总值增长速度逐年加快，由１９９９年的７．５％提升到２００３年的１２．５％，年均递增９．２％。结构调整步伐加快，经济运行质量显著提高，三次产业比例由１９９８年的３８．７∶２８．３∶３３．０调整为２００３年的２９．５∶３２．７∶３７．８，二产首次超过一产。传统农业开始向现代农业转变，经济发展重心向第二产业稳步迈进，第二产业在经济增长中的贡献率达到５３．３％，比１９９８年提高８．３４个百分点。２００３年全市实现财政总收入为１９９８年的１．５７倍，年均递增９．４％；地方财政收入比１９９８年增加４．８４亿元，工商税收占地方财政收入增加额的７０％，税收的增长已明显快于非税收入的增长。

　　农业结构调整稳步推进。２００３年农业占ＧＤＰ比重比１９９８年下降９．２个百分点，但增加值比１９９８年增长１５．３％。在稳定粮食生产的同时，农业结构调整力度加大，经济作物特别是果业、商品蔬菜、草食畜禽和特种水产、苗木花卉四大主导产业不断发展壮大，四大主导产业和特色产业，占农业经济的比重已超过一半以上。脐橙产业发展势头强劲，脐橙面积达７５万亩，产量达１３．３９万吨。农业产业化水平不断提升，全市龙头企业达１３２０家，其中年产值千万元以上的９６家，上亿元的９家，带动农户８５万户。全市形成了１７个重点农产品加工基地，建成了１１２个优质、高效的规模化农产品生产基地。农产品商品率达到６７．４％。

　　工业经济持续较高增长。２００３年全市工业增加值比１９９８年增长７２．４２％。规模以上工业企业实现销售收入比１９９８年翻一番，年均递增１４．８６％；利税总额为１９９８年的９．０９倍，年均递增５５．５％。三年主攻工业成效明显，以稀土和钨为主的有色冶金及新材料、以烟酒为主的食品工业和制药三大支柱产业在工业经济中的地位日益突出，２００３年实现销售收入、利税分别占全市规模以上工业总量的５２％和６４％，比２０００年增长８１．７５％和１７５．４５％。重点企业实力明显增强，２００３年４０户重点企业实现销售收入和利税分别占规模以上工业总量的４７％和６６．７％，其中销售收入过亿元的企业２４户，比２０００年增加１６户；利税超千万元的２２户，比２０００年增加１３户。工业园区建设初具规模，到２００３年，全市１９个工业园实际到位资金１１７亿元，入园企业１１００户，累计实现销售收入１３３．５亿元，实交税金４．８亿元，安置就业１３．２３万人。

　　第三产业快速发展。２００３年第三产业实现增加值比１９９８年增长７４．２％，年均递增１１．７％。旅游业不断壮大，“红色故都”、“客家摇篮”、“江南宋城”、“东江源头”四大旅游品牌开始形成，一批较大规模的综合性旅游景区加紧建设，２００３年国家旅游局批准赣州市为中国优秀旅游城市。五年来，共接待国内游客２３８２．４５万人次，年均递增１０％；国内旅游收入１１８．６７亿元，年均递增１１％；接待海外游客１８．８４万人次，年均递增１０％；创汇４６５５．１９万美元，年均递增１８％。城市社区服务功能不断增强，全市社区服务组织达３．６万个，社区服务实体达２０００多个。市场体系建设不断完善，２００３年全市商品交易市场达６２６个，建设总面积３０６万平方米。城乡市场繁荣活跃，２００３年全市社会消费品零售总额比１９９８年增长５６．５７％，年均递增９．４％。金融业稳步发展，全市金融机构２００３年末存、贷款余额达到３７０亿元和２３６亿元，分别比１９９８年增长１１２．６％和５４．５％。

　　——过去的五年，是基础设施日臻完善、城乡面貌大为改观的五年。五年累计完成全社会固定资产投资４５２．０３亿元，年均递增２０．３％，其中近三年投资３３７．６２亿元，占五年投资总额的７５％。五年基本建设投资累计达１７５．８３亿元，是１９４９至１９９８年４９年的２．０７倍。基础设施建设迈上新台阶。国、省道竣工里程达１３００公里，完成了３２３线、２０６线、３１９线、昌厦公路、杨梅渡大桥、梅林大桥改造或新建；赣定高速公路建成通车里程１００公里，实现了高速公路零的突破；建成县乡村水泥（油）路２９８３公里，乡镇通水泥（油）路率达８６％，行政村通公路率达９６．４％；京九铁路复线赣南段建成通车；赣龙铁路年底即将建成通车。城乡电网改造成效明显，五年累计完成改造投资２２．３５亿元，２２０千伏主变容量增长１３３．３３％，新增线路３９４．１千米，增长１４５．３８％；１１０千伏主变容量增长１２７．２％，新增线路２０３千米，增长１７．１１％。水利基础设施建设投入加大，建设供水工程２２６处、堤防工程１３６．５６公里，加固各类病险水库２２４座；新增节水灌溉面积１６．８万亩，旱涝保收面积５．７４万亩；治理水土流失５００多万亩，净减少水土流失面积２１０万亩；建成内河三级航道９４．８公里，五级航道７２公里。邮电通讯加速向现代化迈进，电信电话用户数比１９９８年增加５倍，移动通讯实现了由模拟到数字的转变。５０万伏输变电工程、三水厂、赣丰线公路改造等一批基础设施项目正在建设之中。

　　城市建设取得新突破。城市化率由１９９８年的１４．５％提高到２００３年的２６．９％。全市新增城市道路２８８．０８万平方米，新增日供水能力４．３万吨，城市建成区面积扩大至１７１平方公里，中心城区建成区面积扩大至３６．９５平方公里。中心城区大力实施了路桥工程、地下管网工程、亮化工程和绿化工程，建成了滨江大道、京九大道、黄金广场、章江南岸、市政中心、体育中心等２０个重点工程项目。以４０个示范镇和一批中心村建设为重点，全市村镇建设快速推进。

　　——过去的五年，是改革开放纵深推进、发展活力大为增强的五年。国企改革攻坚取得重大进展，全市国有企业改制面达８９％，其中工业企业改制面８４．３％，置换职工身份４８９５２人，处置债务２２．７８亿元。所有制结构发生重大变化，非公有制经济特别是个体、私营经济快速发展，２００３年个体、私营经济实现工商税收占财政收入的比重达３２．４％，非公有制经济占经济总量３９％，比１９９８年提高１３个百分点。

　　市县乡政府机构改革稳步推进。市级党政机构由５２个精简为４４个，内设机构由５２８个精简为４６７个。县级党政机构平均由３４个精简为２５个，市县乡三级行政编制分别精简１２％、１７％和２４．４％。

　　农村改革成效明显。２００２年初我市开始启动农村税费改革，农民负担明显减轻，全市农民减负总额达２．６２亿元，减负率达４８．４％，人均减负４１．５元；其他各项配套改革人均减负４４．６元。撤并农村中小学校２４３所。大力推进土地经营权流转，集约化经营水平提高。户籍制度改革不断深入，农村城镇化进程加快。

　　事业单位改革有序展开。市文化系统所属公司以产权制度改革为重点，加快了国有资本的退出。广电系统实行了台网分离。卫生系统有３０多家县乡医疗机构进行了产权制度改革。社会力量办学步伐加快，部分学校实行了股份制改造，以县为主的农村义务教育管理体制得到巩固和完善。市直林业系统下属８家事业单位实行了自收自支。市直建筑、林业、水电交通等勘察、设计单位全部转为企业化管理。科技体制改革不断推进，全市民营科技企业和民办科研机构发展到１００多家。医疗保险制度改革趋于完善，１８个县（市、区）不同程度地启动了基本医疗保险制度改革，参保人员１８．１８万人，基金征缴率９５％。投融资体制不断创新，非国有投资占总投资的３０％以上。财政体制改革继续深化，向构建公共财政体制框架迈出重要步伐。公路管理、粮食购销、商贸流通、供销合作、住房制度、电力体制等方面的改革稳步展开。

　　开放型经济发展不断加快。五年实际利用外资１０．０４亿美元，年均递增３２．４％；实际引进内资３１６．２４亿元，年均递增５５％。引进的工业项目尤其是大项目增多，招商引资质量提高。对外贸易进一步趋好，从２００２年开始扭转了外贸出口连续三年下降的态势，当年比上年增长５０％，２００３年外贸出口达１．３１亿美元，增长５３％。五年累计实现外贸出口４．８８亿美元。

　　——过去的五年，是社会事业全面发展、人民生活大为改善的五年。科技创新能力增强。五年市级以上科技成果１５７项，国际领先水平８项，国际先进水平１８项，获省级以上科技奖励１５项，累计取得专利成果４３９项。高新技术产业不断发展，全市省级高新技术企业１４家，生产高新技术产品的企业２９家，高新技术产品１６５项。科技攻关能力增强，启动了“数字赣州”工程，争取到“技术标准试点城市”、章贡区“可持续发展实验区”等多项省、部级重大科技项目。

　　教育事业加快发展。“普九”教育得到巩固提高，全市有１７个县（市、区）通过“普九”验收。义务教育保障机制初步建立，小学适龄儿童入学率稳定在９９％以上，初中毕业生升入高中阶段学校的比例由１９９８年的３０．３％提高到２００３年的５８．３％，普通高中招生由１９９８年的１．３５万人扩大到２００３年的４．３２万人，在校高中生达１０．９２万人，为１９９８年的３倍。累计筹措资金７．０１亿元，新建和改建中小学校舍１５６．５６万平方米。民办教育快速兴起，全市有民办幼儿园８５２所、民办学校７０所，民办学校在校生３．１７万人。职业教育发展良好，省以上示范性职校１１所，涌现了一批省、部级重点专业。教育结构布局调整日趋完善，教学质量稳步提高。高校办学规模扩大。人才队伍迅速壮大，各类人才总量增加到１４．７２万人，年递增５％。

　　文化卫生等社会事业进一步繁荣。全市组织参加全国、全省各类艺术比赛５０多项，获国家级奖和省级一等奖３０余项（次）；以广场文化、社区文化、特色文化村为载体的群众性文化活动丰富多彩。基本完成广播和电视“村村通”任务，市至县建立了广电光缆传播干线网，广播和电视人口综合覆盖率分别为９２％、９４％，比１９９８年提高１０和８个百分点。医疗卫生事业不断发展，市、县两级都成立了突发公共卫生事件应急指挥机构和办事机构，全市疫情报告网络基本建立，疾控机构国债建设项目稳步实施；重点传染病防治工作进一步加强，传染病发病率继续降低；新型农村合作医疗试点工作进展顺利，城市社区卫生服务步伐加快，公民无偿献血工作富有成效。群众性体育蓬勃发展，圆满完成全民健身计划第一期工程，竞技体育水平逐步提高。坚持不懈地做好人口和计划生育、保护资源、保护环境的工作，可持续发展战略得到较好实施。民政、审计、物价、统计、监察、人防、水文、气象、药品监督与管理、防震减灾抢险、国防动员与后备力量建设、民族宗教、外事侨务、地方志、老龄、妇女儿童等各项工作都取得了较大成绩。

　　人民生活大为改善。城镇居民人均可支配收入年均递增１１．４％，农民人均纯收入年均递增１．８％，城乡居民储蓄存款余额由１９９８年的１３２．０６亿元增加到２００３年的２６８．５９亿元。城乡居民家庭恩格尔系数为３９．６％、５１．０８％，分别比１９９８年下降７．８９和５．６个百分点。城镇居民人均住房面积由１２．５３平方米增加到２７．４２平方米。就业和再就业优惠政策得到全面落实。再就业累计投入资金１．２４亿元，其中地方财政安排６２３７万元。五年新增城镇就业１３．８万人，国有企业下岗职工实现再就业１２．１万人，再就业率为７１．６％。“两个确保”、“一个低保”得到巩固提高。全市下岗职工基本生活保障资金支出４．５９亿元，其中地方财政安排１．６８亿元，占总支出的３６．６％；下岗职工基本生活费足额发放率保持１００％。养老金支付率由１９９８年的７１％提高到２００３年的１００％，养老保险覆盖面从１９９８年的１８．８万人增加到２００３年的２３．７万人。城市居民最低生活保障步入规范化轨道，全市１１．４３万人享受城市最低生活保障。农村特困群众社会救助制度初步建立，２１．４３万人被确定为救助对象。部分企业军转干部的生活困难基本得到解决。扶贫开发深入进行，贫困人口减至２２万，比五年前减少１７万人。

　　——过去的五年，是精神文明和民主法制建设不断加强、发展环境大为优化的五年。以讲文明、树新风为主要内容的精神文明创建活动蓬勃开展，公民道德、职业道德、家庭美德教育深入进行，城乡文明水平和公民思想道德水平明显提高。为期五年的“建设京九文明走廊”活动，在京九沿线建成４个精神文明活动中心、２８个宣传文化站、２８０个农村文化大院。城市创建活动广泛开展，中心城区先后获得了国家卫生城市、优秀旅游城市、综合治理先进城市等一系列称号。坚持并完善了向人大报告工作、向政协通报情况制度，自觉接受人大法律监督和工作监督，重视发挥政协的政治协商、民主监督和参政议政作用，认真办理人大代表意见建议和政协委员提案，提高了政府决策科学化、民主化水平。政府法制建设取得可喜进展，各级领导依法行政观念进一步增强，行政执法、行政复议等工作走上了法制化轨道。普法教育、基层民主政治建设不断深入，社区建设和村民自治扎实推进。强化社会治安综合治理，“严打整治”、“扫黄打非”和揭批“法轮功”邪教组织斗争取得重大胜利，治安秩序保持良好。安全生产状况总体趋好，安全事故起数和死亡人数呈逐年下降趋势。完善信访工作领导责任制，实行严格的信访工作责任追究制度，健全信访工作网络，及时了解掌握并妥善解决了群众反映的实际问题，政治安定、社会稳定的局面得到巩固和发展。

　　经济发展环境不断改善。行政审批制度改革取得明显成效，市政府各部门、市属各单位共取消行政审批事项４５２项，取消率达６９．２％。积极推进政务公开，凡市政府在制订出台重大决策之前，都广泛征求社会各界的意见，２００３年市政府本级有１６６个文件登刊登报，政务公开面达９４．８３％，实施政府部门办事承诺制度，有６４个市直单位作了公开承诺。在全省率先设立了综合收费办证服务大厅，将所有涉及收费办证的４５个综合管理和行政执法部门的２１９项收费、办证业务全部集中在一个大厅内，实行“一站式”服务。

　　２００３年，各级政府认真学习贯彻邓小平理论和“三个代表”重要思想，以加快发展为主题，以改革开放为动力，以结构调整为主线，努力克服发展中的矛盾和问题，特别是经受住了“非典”、洪涝及严重干旱三大严峻考验，圆满完成了一届人大五次会议确定的各项目标任务，经济建设和社会事业取得了显著的成绩。全年实现生产总值３４４．８２亿元，比上年增长１２．５％，创１９９７年以来的新高。实现财政总收入３１．４亿元，增长２０．６％，为五年以来的最高增速；财政收入占ＧＤＰ的比重达到９．１％，比上年提高０．７个百分点；实现地方财政收入１９．６８亿元，增长１３．５％。规模以上工业企业实现销售收入突破１００亿元大关，达到１１１．１亿元，增长３３．５５％；完成工业增加值３０．５５亿元，增长２３．８３％；实现利税１３．４６亿元，增长５７．４％。实现农业总产值１５４亿元，增长３．１％。城镇以上完成固定资产投资１４４．３１亿元，增长５７．４％，其中基本建设投资９３．２３亿元，增长９７．５％。实际利用外资４．７６亿美元，增长１０２．９％；实际引进内资１６８．９５亿元，增长１．３５倍；外贸进出口总值２．３１亿美元，增长７３％。个私经济实现工商税收１０．１７亿元，增长２９．１％。实现社会消费品零售总额１２０．１４亿元，增长１０．９％。城市居民人均可支配收入６７２３元，增长８．９％；农民人均纯收入２２４０元，增长２．３％。全市金融机构各项存、贷款余额分别比年初增长１９．３％和２２．１％。全年新增从业人员３．８７万人，国有企业下岗职工再就业１．９８万人，省外劳务输出８９万人，城镇登记失业率３．７％。人口自然增长率８．５１。其他各项社会事业也取得了新进展。

　　各位代表，过去五年取得的成就，是全市人民同心同德、励精图治、艰苦奋斗的结果，是社会各界大力支持和通力合作的结果。在此，我谨代表市人民政府，向全市工人、农民、知识分子和干部，向所有社会主义劳动者和建设者，向驻市人民解放军、武警官兵、公安干警，向各民主党派、工商联、各人民团体和各界人士，向中央和省驻市单位，向所有参与、关心和支持赣州发展的港澳台同胞、海外侨胞，表示衷心的感谢和崇高的敬意！

　　回顾过去五年的奋进历程，我们倍感取得的成就来之不易，积累的经验弥足珍贵。总结五年的工作实践，我们的主要体会是：

　　（一）始终坚持围绕中心、紧扣主题，把加快发展作为富民兴市的第一要务。对于赣州这样的经济欠发达地区和革命老区，无论是解决目前存在的困难和问题，还是缩小与发达地区的差距，都必须加快发展。五年来，我们坚持以经济建设为中心不动摇，咬住发展目标不放松，努力拉高标杆、高点定位，力求以世界眼光和战略思维审视市情，把握经济全球化和区域经济一体化的大趋势，不断深化对加快发展的认识，丰富加快发展的内涵，完善加快发展的思路。始终坚持把经济工作放在首位，把解放思想、加快发展置于万事之先，贯穿于五年政府工作的全过程。每年在市政府机关及各部门、各单位开展以发展为主题、以转变作风为重点的大学习、大讨论、大整顿活动，不断摒弃不思进取、妄自菲薄、悲观失望、怨天尤人的思想情绪，大力弘扬自强不息、励精图治的精神，增强抢抓机遇、攻坚克难的信心，树立后来居上、抢位争先的壮志，着力营造集中精力抓经济、你追我赶争发展的浓厚氛围。从赣州的实际出发，充分发挥比较优势，积极探索一条具有时代特征、老区特点、赣州特色的发展路子，努力为先进生产力发展开辟道路，推动全市经济实力和综合竞争力不断迈上新台阶。实践告诉我们，只要以富民兴市统揽全局，矢志不渝地用发展的目标凝聚人心、鼓舞干劲，用发展的办法解决前进中的困难和问题，用发展的业绩评判是非得失，用发展的成果造福于人民，我们就一定会实现赣州崛起的宏图伟业！

　　（二）始终坚持与时俱进、开拓创新，把改革开放作为加快发展的强大动力。加快发展的根本出路在于改革开放。五年来，我们以创新的思维、胆略和举措，不断冲破一切妨碍发展的思想观念，改变一切束缚发展的陈规陋习，革除一切影响发展的体制弊端，以市场化改革为取向，纵深推进经济体制和机制创新，推动投资主体多元化、经济成份多样化、运行机制市场化。围绕国有企业改革这个中心环节，以产权制度改革为重点，全面加速国有产权和职工置换身份的改制，通过股、售、转、卖、并、破等多种改革方式，积极组织国有资本从竞争性领域退出，鼓励和引导非公有制经济有序进入。盘活了一大批国有存量资产，吸纳了一大批民营资本，引入了一大批外来资金，形成了国有老企业活力重现、民营企业脱颖而出、外资企业迅速发展的良好态势。在政府宏观管理层面上，从转变职能入手，紧紧围绕深化行政管理体制改革，精简政府机构，减少审批事项，下放管理权限，创新管理方式，提高政府效能，增强了经济发展的内生动力。

　　以大开放为主战略，坚持对外开放与对内放活相结合，“走出去”与“引进来”相结合。不断加大招商引资力度，建立和完善招商引资责任制，落实目标任务，强化考核激励，形成了领导带头、上下联动、部门配合、各行各业共同参与的招商引资工作机制。不断创新招商引资方式，以小分队招商为主，驻点招商、网上招商、委托招商、以友招商、以商招商、大型活动招商等多形式并举，降低了招商成本，提高了招商效果。不断加强对外经济技术交流与合作，开放型经济的水平得到进一步提升。实践告诉我们，改革开放是加快发展的强大动力，什么时候改革开放力度大，什么时候发展就快；哪个地方改革开放办法多，哪个地方变化就大。

　　（三）始终坚持扩量提质、夯实基础，把结构调整作为提升经济发展水平的主线。赣州经济相对落后，根源在工业化水平低。五年来，我们紧紧围绕结构调整这条主线，以工业化为核心，抓好经济结构的战略性调整。特别是自２００１年起，全力实施三年主攻工业战略，打了一场做大做强赣州工业经济的攻坚战。突出重点培育支柱产业，加大了产业整合和资源集约化配置力度，注重市场营销和创立品牌，强化融资和新产品研发，支柱产业整体实力明显提高。实施扶优扶强战略，集中精力扶持重点企业，一批重点企业已经成为工业经济快速发展的主要支撑力量和领头羊。花大力气抓好重点项目，引进发展了一批拉动作用明显、带动力强的大项目。下功夫开发建设园区。通过财政注资、经营土地、吸纳社会资金、招商引资、市场化运作等手段，完善园区基础设施建设，工业园区正成为承接沿海产业转移的主要平台，成为全市经济发展的重要增长极。

　　“三农”问题事关我市改革发展稳定的大局，任何时候都不能忽视和放松。五年来，我们坚持农业的基础地位不动摇，大力调整和优化农业结构，具有竞争力的特色经济产业快速兴起，农业产业化经营的途径不断拓展。建设了一批优势农产品专业化生产基地，培育了一批农业龙头企业和农产品品牌，壮大了一批农产品专业批发市场，组建了一批农民经济合作组织，农业和农村经济的市场化进程加快。着眼于提高农民生活水平，增加农民收入。树立逐步减少农民才能富裕农民的理念，进一步打破农民进城经商务工的不合理限制，农村人口向非农产业和城镇转移就业的步伐加快。着眼于改善农业生产条件，加大对农业的投入，农田水利设施继续改善，农业服务体系日趋健全，农业科技创新能力不断增强。实践告诉我们，统筹城乡经济社会发展，把解决“三农”问题作为政府工作的重中之重，大力加强农业，发展农村经济，增加农民收入，是全面建设小康社会的关键所在。

　　（四）始终坚持加大投入、完善功能，把加强基础设施建设和推进城市化进程作为增强区域竞争力的突破口。在经济和社会发展中，必须善于抓住和解决主要矛盾，才能取得事半功倍的效果。五年来，我们从基础设施建设这一全局性、长远性的工作着眼，从投入不足这一最具牵动性、突破性的主要矛盾着手，抓住国家扩大内需，实施积极财政政策的机遇，努力争取国债资金和专项资金，大力招商引资吸纳国内外资金，积极从体制机制上激活民间投资，充分挖掘市场融资潜力，不断加大资本运作和筹资力度，多渠道、多方位、多层次破解资金难题，扩大投资规模，加强基础设施建设，办成了一些多年想办而没有办成的大事，集中力量建成的一大批基础设施项目，有力地推动了全市经济和社会发展步入“快车道”。如果没有五年来加大投入的决心和前所未有的资金投入量，就不可能有基础设施建设的骄人业绩，也没有促进经济快速增长的强劲拉动力，更无从谈起经济和社会发展后劲的持续增强。

　　城市化是走向现代化的必由之路。五年来，我们着力抓好规划、建设、管理和经营城市四大环节。在城市规划上，２００３年中心城区发展规划编制进行了国际招标，绝大多数县（市）已完成或正在进行新一轮总规修编工作。在城市建设上，加大了新城区中心路网、排水排污工程及供电、通讯、供水系统建设力度，实施了一批绿化美化亮化净化项目，中心城区的综合功能得到提升。两个次中心城市和各县（市）城关镇建设全面推进，在扩大规模、完善功能、提高品位、优化环境等方面取得明显进展。在城市管理上，进一步深化城市管理体制改革，市容环境状况得到改善。在经营城市上，建立土地收购储备制度，实行经营性土地使用权招标拍卖挂牌出让，充分利用城市土地资源做好经营土地的文章，激活变现各类城市资产，形成了财政、金融、社会相结合的多元化筹资机制。实践告诉我们，牢牢抓住基础设施建设和推进城市化这个突破口，着力提高资源优化配置程度和人口聚集程度，扩大经济发展容量，改善经济发展环境，才能不断增强区域综合竞争能力，加速赣州的现代化建设进程。

　　（五）始终坚持统筹兼顾、协调发展，把实施可持续发展战略作为推动社会全面进步的长期任务。做好人口、资源和环境保护工作，是实施可持续发展战略的必然要求。五年来，我们坚持党政一把手亲自抓、负总责，建立和完善人口与计划生育目标管理责任制，巩固和健全计划生育工作网络，全面完成了各年度人口控制计划，人口增长速度得到控制，人口出生率、自然增长率连年下降，全市总体进入了低生育水平。

　　加强资源保护和合理利用。坚持在保护中开发、在开发中保护的方针，以保护耕地为重点，严格土地审批，制止和纠正各类“圈地”和滥占耕地行为，规范土地市场秩序，基本做到耕地占补平衡。持续开展矿产资源管理秩序的治理整顿，关闭了一批非法开采的矿点，整治了一批“五小”企业。抓住钨和稀土这两个优势矿种，在有效保护和充分利用上做文章，创造了国家保护性开采特定矿种的“四统一”模式，乱采滥挖现象得到遏制。

　　加大环境保护力度。严查环境违法行为，严格老污染源整治，深入持久地开展城市废水、废气、废物和噪音污染的治理，着力改善城市环境质量，城市环境综合整治定量考核连续多年取得较好成绩。加强生态保护，农业生态环境进一步优化，农村水土流失得到遏制，自然条件、人居环境有了改善。实践告诉我们，绝不能以牺牲环境和浪费资源为代价求得一时的经济发展，必须高度重视经济效益、社会效益和生态效益相统一，高度重视经济与人口、资源和环境相协调，高度重视社会的全面进步和人的全面发展，这是我们必须始终坚持的重要指导思想。

　　（六）始终坚持求真务实、执政为民，把维护和实现最广大人民的根本利益作为一切工作的出发点和归宿。加快赣州经济社会发展，必须努力建设廉洁、勤政、务实、高效的政府。五年来，我们紧紧围绕市委和市人代会确定的目标任务，树立政府工作一盘棋思想，坚持整体部署、阶段推进、团结协作、务求实效。注意眼睛向下，足行基层，深入实际调查研究，及时掌握真实情况，集中群众智慧，提高政府决策的科学性、民主性和可操作性。注意讲究工作效率和办事效果，对政府主要工作进行定期督查，对重点工程进行现场办公，对难点问题进行专题协调，对看准了的事，雷厉风行、说干就干，努力做到工作快节奏、高效率，办事言必行、行必果。坚持依法行政、依法办事、从严治政、廉洁自律，纠正部门和行业不正之风，坚持不懈地开展反腐败斗争，加强和改进政府工作。

　　群众利益无小事。五年来，我们时刻把人民利益放在心中，把实现人民的利益作为政府的神圣使命，躬身践行为人民服务的宗旨，想问题、定政策、办事情，着眼于广大人民群众，竭尽全力让人民群众得到应该得到的实惠。我市城乡居民收入较低，困难群体较多，我们切实正视并努力解决事关人民群众重大利益的难点、热点问题，帮助城乡低收入阶层和贫困居民排忧解难。加强社会保障体系建设，加大财政对社会保障的投入，建立再就业安置基金，实施城镇居民最低生活保障制度，建立多层次的社会救助制度，广泛开展扶贫帮困活动。努力为人民办实事、办好事，认真解决城市就业、就学、就医、出行、住房和农村用电、用水、道路交通等与人民群众利益密切相关的问题。高度重视“非典”防疫工作，确保无一人感染“非典”。正确处理改革发展稳定的关系，把发展的速度、改革的力度和社会可承受的程度有机统一起来，兼顾各方面利益，认真细致地做好改制企业职工安置、征地拆迁补偿、企业和农民减负等工作，在社会稳定中推进改革与发展，在改革与发展中促进社会稳定。实践告诉我们，只要沉下心来干事业，扑下身子抓落实，关注民生、以民为本，切实把人民群众的利益维护好、实现好、发展好，才能不辱使命、不负重托，才能无愧于历史、无愧于时代、无愧于人民！

　　在肯定成绩的同时，我们清醒地看到，发展中还存在不少矛盾和问题，政府工作也有许多不足。主要是：经济总量偏小，经济实力较弱，发展速度偏低，与全省平均水平相比仍有较大差距。工业化、城市化滞后，支撑带动和集聚辐射能力不强，经济结构性、素质性矛盾比较突出。影响经济发展的体制性、机制性障碍尚未消除，经济社会发展环境有待进一步优化，市场取向的改革任务艰巨。就业再就业形势严峻，社会保障能力薄弱，落实“两个确保”、“一个低保”的压力依然很大。农民收入增长缓慢，部分群众生活仍较困难。政府职能转变不能完全适应形势发展的需要，个别政府部门及少数工作人员存在服务意识不强、作风不实、效率不高、为政不廉等现象。对这些问题，应引起高度重视，在今后的工作中努力加以解决。

　　二、今后五年的工作建议

　　各位代表，前不久胜利召开的市第二次党代会，为我市经济和社会发展绘就了宏伟蓝图，确立了基本方略，明确了目标任务，制定了战略举措，赣南这块红色的土地，必将呈现出一幅壮美而绚丽的新画卷。今后五年是我市全面实施“对接长珠闽、建设新赣州”发展战略，在全省率先完善社会主义市场经济体制的关键时期。面对各地你追我赶、竞相发展的竞争态势，我们务必居安思危、审时度势，增强发展的责任感和紧迫感，以饱满的热情，昂扬的姿态，进取的精神，扎实的工作，再创赣州新的辉煌。

　　今后五年政府工作的指导思想是：以邓小平理论和“三个代表”重要思想为指导，深入贯彻党的十六大和十六届三中全会精神，按照市第二次党代会的战略部署，以在全省率先完善社会主义市场经济体制、促进经济社会全面发展为总目标，树立和落实科学的发展观，全面实施“对接长珠闽、建设新赣州”发展战略，加大经济结构调整力度，加快改革创新步伐，大力发展开放型经济，不断提高工业化、农业产业化和城市化水平，协调推进三个文明建设，加速区域性现代化中心城市建设进程，为实现全面小康社会奠定坚实的基础。

　　主要奋斗目标是：

　　——基本形成与人口大市相适应的较强经济实力。经济发展总量和质量明显提升，持续发展能力和城市综合竞争能力明显增强。到２００８年，全市生产总值跃上６００亿元台阶，综合经济实力在全省名列前茅；三次产业结构进一步优化，二产比重超过４０％，财政总收入五年翻一番；城镇人口失业率控制在３％以下，城乡居民可支配收入年均分别增长８％和５％以上，人口自然增长率控制在８．２‰以内。

　　——基本形成完善的社会主义市场经济体制和更具活力、更加开放的经济体系。全面实现与“长珠闽”在体制机制上的融合，在全省率先完善社会主义市场经济体制，经济社会发展的体制活力和内生动力不断增强，政府成为营造环境的主体，市场成为资源配置的主体，企业成为市场竞争的主体，民众成为创造财富的主体的局面加速形成。

　　——基本形成以中心城区为龙头、次中心城市为节点、重点镇为基础的城镇发展构架。全面拉开中心城区新框架，构筑以中心城区为圆心的一小时都市经济圈，瑞金、龙南两个次中心城市的规模和功能明显提升，重点镇建设取得突破性进展。到２００８年，全市城市化率达到４０％以上，中心城区达到７０平方公里和７０万人口，成为国家园林城市；到２０１５年中心城区达到１００平方公里和１００万人口，成为文化内涵丰富、生态环境优美、城市功能完善、交通运输便捷、产业经济集聚、辐射带动力强、最适合人们居住和创业发展的赣粤闽湘四省通衢的区域性现代化中心城市。

　　——基本形成功能齐全、综合配套的基础设施网络。加快构筑迅捷流畅的大通道，形成“十”字型铁路网、“两纵两横”高速公路网、“三纵三横”干线公路网，扩大机场吞吐量，提高通航能力，全方位构建与“长珠闽”特别是珠江三角洲和闽南三角区一体化的立体交通网络，全面解决支撑我市经济建设的电力能源等“瓶颈”问题，完善配套各类公共服务设施，全面提升赣州的“对接”功能和经济发展的承载能力。

　　——基本形成以人为本，全面、协调、可持续发展的格局。把科学的发展观贯穿于经济发展的全过程，实现赣州古城历史文化底蕴与现代都市文明优势相融合，经济建设与社会事业协调发展，城乡差距明显缩小，生态环境显著改善，三个文明齐头并进，人与自然和谐共生，区域发展优势和统筹整合水平大为提升。

　　在今后五年的工作中，必须牢牢把握以下五点：

　　一是必须强化集约发展、均衡发展、可持续发展和一切为民谋发展的新理念。顺应经济社会发展的内在规律，更加注重集约发展，提高资源配置效率，转变经济增长方式，促进经济结构进一步优化。更加注重均衡发展，调整城乡二元结构，坚持以城带乡、以工促农、城乡互动、协调发展。更加注重可持续发展，统筹人口、资源、环境与经济、社会协调发展。更加注重一切为民谋发展，以民富、民享为基本取向，使赣州人民共享改革发展的成果，得到更多的实惠。

　　二是必须把握“对接长珠闽、建设新赣州”发展战略的新内涵。重点加快与“长珠闽”在体制机制上的对接，围绕建立和完善十大体系，全面提高市场化运作水平；加快与“长珠闽”在城市环境和基础设施上的对接，围绕中心城区、“一小时都市经济圈”、二个次中心城市和基础设施四大建设重点，打造与“长珠闽”特别是珠三角地区对接的新平台；加快与“长珠闽”在产业发展上的对接，围绕形成以赣州中心城区为核心增长极、以工业为支柱的开放型经济新格局，大力促进内生型经济与开放型经济的融合，实现与“长珠闽”特别是珠三角地区经济的一体化。

　　三是必须开拓新型工业化、农业产业化和城市化协同推进的新思路。努力形成“三化”联动的良好效应，在走新型工业化道路的同时，运用工业的理念推进农业产业化建设，围绕主导产业发展农产品加工业，围绕农业龙头企业发展城镇服务业，加快农村剩余劳动力转移，通过工业化和城市化，促进农村的现代化，最终解决“三农”问题。坚持工业化和城市化“双轮驱动”，依托城镇发展工业园区，引导乡镇工业向中心城镇集聚，企业向工业园区集聚，以培育产业集聚推进城市建设，通过繁荣城市经济带动农村经济，加快农村工业化、城市化进程。

　　四是必须构筑体制创新和扩大开放的新高地。以在全省率先完善社会主义市场经济体制为目标，坚定不移地推进各项改革，强化制度建设和创新，着力形成微观和宏观两个层面始终充满活力的体制优势，使赣州真正成为创新之城、活力之市。全面实施大开放主战略，以对接“长珠闽”为契机，积极主动融入世界经济体系，在更大范围、更广领域、更高层次上参与国内外经济技术合作和竞争，借助外力，加速推进我市现代化进程。

　　五是必须增创经济社会发展环境的新优势。按照建设“安全、文明、诚信、高效”发展环境的要求，加紧建立健全社会信用体系，进一步营造良好的社会信用环境；加大完善市场体系和规范市场秩序力度，进一步营造良好的市场环境；加快依法治市进程，进一步营造良好的法治环境；切实转变政府职能，进一步营造“亲商、安商、富商”的投资环境，以良好的环境树立形象、吸引投资、留住外商、提升人气，最终赢得大发展。

　　２００４年是我市实施“对接长珠闽、建设新赣州”发展战略的第一年，也是新一届政府施政的开局之年。做好今年的工作，对于实现今后五年的目标，意义重大。２００４年全市经济和社会发展的预期目标为：生产总值增长１２％，全社会固定资产投资增长２０％，社会消费品零售总额增长１０％，居民消费价格总水平上涨幅度控制在３％左右，财政总收入增长１５％，实际利用市外资金增长３０％，城镇居民人均可支配收入增长９％，农民人均纯收入增长４．５％，城镇登记失业率控制在３．３％以内，人口自然增长率控制在８．２以内。

　　完成上述目标任务，今年要突出抓好以下几方面工作：

　　（一）加快农业产业化进程，促进农村经济全面发展

　　坚持“多予、少取、放活”的方针，以增加农民收入为目标，全面贯彻落实今年中央、省两个一号文件的各项惠农政策和市２８条兴农措施，农业税降低３个百分点，鼓励农民种粮的补助资金必须全部到位。高度重视粮食生产，主攻单产，提高质量，努力扩大种植面积，防止土地撂荒，切实提高粮食综合生产能力。在确保粮食安全的前提下，坚定不移地调整、优化农业结构。大力发展高效优质经济作物，发展标准化、无公害化、绿色化生产，提高农产品品质。在确保基本农田不减少的前提下，坚定不移地推进农业规模经营。继续大力发展果业、商品蔬菜、草食畜禽与特种水产、苗木花卉四大主导产业。抓住脐橙开发管理不放松，每年新开发高标准脐橙园１５万亩左右。尽快形成“一县一业、一乡一品”的农业区域布局。在巩固农业基础地位的前提下，坚定不移地推进农村工业化和城镇化。以工业化理念推进农业的发展，培育壮大一批优势农业龙头企业，扶持引进一批科技含量高、规模大、以出口创汇为主的外向型龙头企业，整合重组一批传统农业龙头企业，支持、引导农村专业合作组织和各类协会发展，提高农业组织化程度，培育发展农村二、三产业，以产业吸引农民进入城镇，提高农业产业化和农村工业化水平。继续采取强有力的措施，切实减轻农民负担。认真落实对农村特困户及重点优抚对象减免农业税政策。继续抓好新阶段的扶贫开发工作，年内减少贫困人口２万人，完成深山区和库区１．２万农村群众的移民搬迁任务。积极开展农村“三清三改”活动，改善农民的生活环境。抓好以水利基础设施建设为主的农村“六小”工程建设，决战４年完成全市２５１座病险水库的除险加固。加强防汛抗旱和防灾减灾工作，不断改善农业生产条件。

　　（二）走新型工业化道路，促进工业经济提速增效

　　按照走科技含量高、经济效益好、资源消耗低、环境污染少、人力资源优势得到充分发挥的新型工业化道路的要求，进一步做大做强赣州工业经济，工业增长高于全市生产总值增长速度８—１０个百分点。围绕中心城区、“一小时经济圈”、两个次中心城市以及赣粤高速和赣龙铁路，重点进行产业布局，着力形成“一区一圈两翼两线”的工业发展格局。抓紧制定并组织实施工业主导产业规划，大力发展六大主导产业，在抓好原有的食品、有色冶金及新材料、制药三大支柱的基础上，培植现代轻纺、机电制造和新型建材三大主导产业，推进产业集中、升级和创新，逐步形成具有区域特色的优势产业集群。将稀土、钨、锡、钴金属及材料、汽车变速箱培育成全国的重要生产基地，将卷烟、酒、制药、造纸、制鞋、服装纺织、新型干法水泥培育成全省的重要生产基地。继续培育５０户重点企业，根据行业不同情况，制定专门措施，通过整合重组和靠大联大等方式，瞄准国内外的大公司、大集团，实施大协作、大配套，以低成本和差别化策略，逐步融入“长珠闽”产业链，进而融入全球产业分工和市场体系。加快企业文化和品牌建设，促进企业围绕市场创名牌，创立名牌拓市场，推动以市场为取向的量的扩张。大力发展深加工和科技含量高的项目，积极策划、包装、新上一批带动力强的大项目。鼓励引导符合条件的企业上市融资。推进企业信息化建设。建立和完善中小企业服务体系。强化工业园区的规划、建设和管理，加快园区设施配套和功能提升，着力把工业园区打造成投资高地和成本洼地。以工业园区为主要载体，大力承接沿海发达地区劳动密集型、出口加工型企业的大规模转移，以产业的聚合和裂变，实现赣州经济规模和经济总量的加速扩张。

　　（三）大力发展城市服务业，促进城乡经济繁荣活跃

　　把旅游业作为支柱产业来抓，搞好旅游规划，加快旅游基础设施及景点建设，努力打造国内外知名的旅游景区。加强与周边地区特别是“长珠闽”的合作，整合旅游资源，建设精品旅游线路。强化旅游促销，加大“红色故都”、“江南宋城”、“客家摇篮”、“东江源头”等旅游品牌的宣传，提高赣州旅游知名度。大力发展商贸物流业，加快建设商业零售大企业和仓储中心，建立与完善一批大型批发市场，积极探索代理、展览和仓储、直销、电子商务、连锁经营等新型业态和流通方式；优化重组现有仓储、码头、铁路等物流资源，加强与国内外大型商贸和物流公司的合作，培育区域性现代物流企业和物流中心，把赣州打造成周边地市重要物资商品集散地。稳步发展房地产业，全面放开搞活住房二级市场。加快信息服务业发展，建设高速、宽带、大容量的信息网络，推进信息技术在各领域的应用。规范和扶持行业协会及会计、律师、审计、工程咨询等中介服务组织，培育产权、资本、技术和劳动力等要素市场。重视发展社区服务业，鼓励社会力量投资社会服务设施及服务网点，探索物业管理公司、居委会、社区服务“三位一体”的新型小区管理体制，建立和完善社区服务网络。

　　（四）深化各项改革，推进体制机制创新

　　以产权制度改革为核心，加大国有经济布局调整和企业改革力度，完成市属国有（集体）企业“两个置换”的扫尾工作，支持和帮助中央、省属驻市企业的改革和重组。深化国有资产管理体制改革，组建市国有资产监督管理委员会、国有资产经营公司和授权经营的国有控股公司。推进公司制改造，加快建立现代企业制度。坚持政事分开、事企分离、有序推进的原则，深化社会事业单位改革，使事业单位尽快走向市场。积极推进农村经济体制改革，巩固和扩大农村税费改革成果，完善户籍、土地、乡镇机构和义务教育体制等各项配套改革。加快投融资体制改革。建立民间投资激励机制，打破行业垄断和部门分割，拓展非国有经济的投资领域。支持市商业银行、农村信用社等地方性金融机构的改革与发展，积极发展投资公司等非银行金融机构，形成地方多渠道融资体系。深化财政体制改革，规范财政预算管理，扩大政府采购范围，优化支出结构，加快建立公共财政体制。进一步推进社会保障、粮食购销、电力、药品流通和公路管理等各方面的改革，激活经济主体，增强发展力和创新力。

　　促进非公有制经济更好更快发展。继续清理涉及非公有制经济发展的政策文件，坚决取消对非公有制经济的歧视和限制，全面落实扶持非公有制经济发展的政策措施，使非公有制经济真正享受国民待遇。建立中小企业信贷担保机构和发展基金，着力解决发展中的融资难问题。鼓励非公有资本进入法律未禁止的基础设施、公用事业等领域，支持非公有制企业参与国有企业改革，加快富有活力的混合所有制经济格局形成。引导非公有制企业转变经营和管理方式，逐步建立现代企业制度，实现制度创新、技术创新、管理创新，提高企业经营管理水平。进一步促进非公有制经济裂变式发展，通过联合、兼并、重组等方式，上规模、上档次、创名牌，走公司化、集团化、国际化之路，增强核心竞争能力。

　　（五）大力招商引资，发展开放型经济

　　做好招商引资的基础性工作。严格落实责任，完善招商引资激励机制，加强专业招商队伍建设，舍得把有知识、有能力、素质高的人才充实到招商引资第一线。围绕赣州产业发展规划，精心编制招商项目，分级建立招商项目库。创新招商引资方法，坚持以商招商，完善委托招商，利用网络招商，依托园区和产业链项目招商。继续实行小分队招商，抽调人员成立专业招商队伍，瞄准重点地区、重点客商、重点产业上门招商；开展系列活动招商，积极组团参加国家与省举办的重大投资贸易洽谈活动，加强与知名企业界人士的联系，组织好各种项目推介会和经贸洽谈会。进一步优化投资软环境。建立健全招商服务机制，全面实施项目代办制、首问责任制、帮办服务制、责任追究等制度。深化行政审批制度改革，最大限度地减少行政审批事项，提高办事效率。着力解决外来投资中商务、事务和企业生产经营中遇到的困难和问题，加大督查、考核和项目跟踪力度，杜绝“三乱”现象，做优做响赣州的环境品牌，吸引更多的客商前来投资创业。

　　扩大对外交流，加强区域合作。根据“长珠闽”及周边城市的功能定位和产业布局，重视利用“泛珠三角”的辐射力和影响力，研究制定区域合作规划，落实具体措施和载体，促进政策协调、规划衔接、产业配套和商品、要素的自由流动，加强科技、文化、商贸、旅游、金融等领域的交流与合作。认真办好世界客属第十九届恳亲大会，加强与世界各地的联系，把赣州推向世界。落实扶持外贸发展的各项政策，努力扩大外贸出口。强化口岸功能，完善海关、检验检疫服务网络，各涉外部门联动，实现“大通关”。

　　（六）加强基础设施建设，增强经济发展后劲

　　今年固定资产投资努力实现１８０亿元。加快交通通讯建设。积极争取赣龙高速公路、赣韶高速公路的开工建设；抓好赣粤高速公路龙南里仁至定南段、赣龙铁路、赣丰线公路改造的续建，抓紧黄金机场搬迁；做好赣韶铁路、赣郴高速公路、瑞金至平远高速公路、赣州火车站扩建等项目的前期准备工作；完成国省道４００公里、县乡村公路８００公里改造；新建城市防洪工程２００公里。抓好无线市话网络优化和宽带城域网等工程建设；加快数字赣州、电子政务网、公众和企业网及其各项配套建设。加快电力基础设施建设。着力抓好瑞金（赣州）电厂、居龙滩电站、吉安至赣州５００千伏输变电工程、１１０千伏和２２０千伏主电网的新建和续建；继续抓好城乡电网改造。加快口岸服务设施建设。完成海关及商检查验场、保税仓库等项目。加强对项目开发的指导、调度和考核，争取今年市级项目库新入项目６００个以上。

　　加快城镇建设。按照规划先于建设的原则，高标准编制好中心城区总体发展规划，以加快章江新区路网工程建设和“三江六岸”工程建设为突破口，拉开中心城区发展框架。抓好中心城区路面、人行道、路灯改造及市政基础设施维护管理，抓紧建设迎接世界客属第十九届恳亲大会在我市召开的一系列工程项目。加快两个次中心城市和县（市）城镇建设步伐，增强要素吸纳、产业集聚、经济辐射功能。切实贯彻执行国家的有关规定，坚持美观大方与节约用地相结合，因地制宜搞好城市建设，制止脱离实际的工程建设项目。加强城市经营管理，树立以经营求发展的理念，建立健全城市建设投入、回报、偿还、再投入的良性循环机制，加大土地收购储备力度，收足用好土地收益。完善城市管理体系，以“城市管理年”为契机，开展创建园林城市活动，抓好市容市貌整治。改革城市公用事业管理体制，逐步使公用事业部门实行建管分开、管养分离，把环卫保洁、垃圾处理、市政养护、园林绿化等公共服务项目推向社会、推向市场，提高城市管理水平。

　　（七）推动社会全面进步，促进三个文明协调发展

　　落实可持续发展的各项措施。做好人口与计划生育工作，完善人口综合治理工作机制和人口与计划生育公共服务体系，建立流动人口以现居住地管理为主的工作机制，稳定低生育水平，提高人口素质；整顿资源管理秩序，贯彻落实中央最严格的耕地保护制度，从严控制用地规模，加强基本农田保护。抓好矿产资源勘查、开采和经营管理，整顿和规范开采秩序，整合矿产资源，促进资源科学合理开发和永续利用。大力发展循环经济，推进节能型社会建设。强化环境保护，重点防治大气、水质、土壤和固体废料等污染。继续抓好“五小”企业整治，淘汰污染严重的落后生产能力。加大森林资源保护力度，加强水土流失治理，搞好封山育林、植树种草和森林防火，适时在全市禁伐天然阔叶树商品材，遏制人为破坏生态和以牺牲环境为代价的急功近利行为。

　　做好就业和社会保障工作。坚持就业优先战略，全面落实各项就业和再就业优惠政策，广开就业门路，想方设法增加就业岗位，努力帮助下岗失业人员，尤其是“４０５０”等就业困难人员实现再就业。搞好劳动力市场网络化、信息化和社区化建设，积极开展劳务输出，加快农村富余劳动力转移。引导广大群众转变就业观念，鼓励自谋职业、自主创业，逐步建立就业和再就业的长效机制，完善就业困难人员和困难群众的政府帮扶、托底机制。完善与我市经济发展水平相适应的社会保障体系，加大对社会保障资金的收缴和支出力度，进一步巩固“两个确保”、“一个低保”和农村特困群体救助工作，搞好“三条保障线”的衔接和并轨，依法扩大养老、医疗、失业等社会保险的覆盖面。

　　提高科技进步和创新能力。深化科技体制改革，加强产学研合作，建立以企业为主体的技术创新体系，引导和扶持企业通过技术创新优化产品结构，提高科技含量。围绕工业六大主导产业和特色农业，着力实施一批产业化项目。加大民营科技企业培育力度，积极引进技术含量高的项目尤其是高新技术成果产业化项目、专利技术项目。抓紧国家“标准化试点城市”和章贡区“社会可持续发展综合试验区”项目的实施，加快“数字赣州”工程建设，在重大科技攻关上取得突破。加大农业科技成果转化与先进适用技术推广力度，抓好赣南现代农业科技示范园建设。对现有市属科研机构进行结构、人才、资产重组，组建赣南科学院。

　　发展多层次教育。坚持农村义务教育“以县为主”的管理体制，完善义务教育保障机制，巩固“普九”成果。多渠道筹措资金，加大校舍危房改造力度，推进中小学标准化建设。大力发展高中教育和职业教育，加快优质高中建设步伐，规范和发展民办教育，鼓励社会力量办学。支持驻市和市属大中专院校优化教育结构，扩大办学规模，提高办学质量。加快教育城建设，积极引进市外高等院校到赣州兴办分院、分校。积极发展幼儿教育、特殊教育、继续教育和现代远程教育。大力实施“农民知识化”、“公民职业化”两大培训工程。重视人才工作，推进人事制度改革，积极培育人才市场，多层次、多形式、大规模开展人才培训，落实人才政策，营造引得进、留得住、用得上、能创业的人才发展环境。

　　全面发展文化、卫生、体育等社会事业。积极发展社区文化、企业文化、校园文化和村镇文化，加大精品创作力度。重视历史文化遗产保护和民间艺术挖掘，推进历史文化名城建设，精心打造“红土地文化”、“宋城文化”、“客家文化”。加强社会公共卫生体系建设，强化政府公共卫生管理职能，建立健全疾病预防控制、突发公共卫生事件、卫生监督、医疗救治和公共卫生信息网络体系。加强药品食品监督管理，提高人民群众就医用药质量。加大科学防病知识宣传普及力度，引导人民群众追求科学、文明、健康的生活方式。开展全民健身运动，提高人民群众身体素质和体育竞技水平。关心支持妇女儿童、残疾人和老龄工作。抓好第一次全国经济普查工作。继续认真做好广播电视、民族宗教、外事侨务、对台、人防、档案、地方志、防震减灾、民政优抚等各项工作。加强国防教育和国防后备力量建设，进一步完善国防动员体系，提高预备役部队建设水平，深入开展“双拥”共建活动。

　　加强精神文明和民主法制建设。以思想道德建设为中心环节，加强社会公德、职业道德和家庭美德教育。开展“八进”社区、职业道德标兵、“美德在农家”等群众性精神文明创建活动。进一步加强和改进未成年人思想道德建设，努力培育“四有”和德智体美全面发展的建设者和接班人。充分尊重人民代表管理国家和社会事务的权利，进一步加强基层政权建设，完善居民、村民自治，扩大基层民主，推行和规范厂务公开、村务公开。深入开展《宪法》宣传教育和“四五”普法教育，重点加强对各级行政领导干部、行政执法人员、青少年和企业管理人员的法制教育，增强全民法律意识，不断提高干部群众法律素质，积极推进依法治市进程。

　　切实维护社会稳定。完善安全生产监管体系，层层落实安全生产责任制，防止重特大安全事故发生，提高应急救援能力，保障人民生命财产安全。重视和加强人民群众来信来访工作，开展“基层信访工作年”活动，加大矛盾纠纷排查调处，妥善处理好与人民群众切身利益相关的各类问题。大力整顿和规范市场经济秩序，打击假冒伪劣行为。加强社会治安综合治理，依法严厉打击各种犯罪活动，继续开展与“法轮功”等邪教活动的斗争，破除封建迷信，坚决扫除“黄赌毒”等社会丑恶现象，保持良好的社会秩序，维护社会稳定。

　　（八）进一步加强政府自身建设，提高依法行政和服务水平

　　今年是新一届政府履行职责的第一年，新形势、新任务对政府工作提出了更高的要求。在推进改革开放和现代化建设的进程中，始终要牢记立党为公、执政为民，切实加强政府自身建设。

　　努力建设责任型政府。按照社会主义市场经济体制对政府的要求，切实转变政府职能，加快从无限责任政府向有限责任政府转变，着力解决政府缺位、越位和错位的问题。进一步理顺政府部门职能，明确职责分工，把该管的事情管住、管好，不该管的事情坚决放开，使政府职能真正转到经济调节、市场监管、社会管理、公共服务上来。加强政府工作责任落实和监督力度，强化政府权力和责任的统一，做到有权必有责，用权受监督，侵权要赔偿。继续实行行政执法责任制、评议考核制和过错责任追究制，一项工作由一个部门主管，谁管的事情谁负责，谁出了问题追究谁的责任，杜绝都管和都不管现象。加强公务员队伍的教育与管理，不断增强公务员的事业心和责任感，把敢负责、抓落实作为品质和素质的集中体现，把求实效、创业绩作为能力和才干的鉴别标准，激励公务员在各自岗位上尽心尽力尽职，把赣州的事业不断推向前进。

　　努力建设服务型政府。牢固树立公仆意识和群众观念，把服务型政府建设落实到各级领导深怀爱民之心，恪守为民之责，善谋富民之策，多办利民之事的具体行动中去。切实转变工作作风，反对形式主义和官僚主义，始终坚持深入群众，倾听百姓呼声，关心群众冷暖，千方百计解决好就业保障、工作居住、环境治安、拆迁安置等方面与群众利益息息相关的突出难题，让普通百姓工作顺心、生活安心。对每年确定的重点工作、重点项目尤其是为民办实事办好事的项目，倾注心力，沉下身去，逐项落实，办实办好。进一步拓宽与广大人民群众的联系渠道，坚持和完善领导定期接待日制度、人民建议征集制度、市长电子信箱制度等行之有效的措施，让人民群众有机会说话、有地方讲理、有人帮助解决问题，切实为人民群众排忧解难。

　　努力建设法治型政府。加强政府法制建设，增强法制观念，提高依法行政能力。增加行政行为透明度，规范政府运作机制，完善决策程序，理顺行政执法体制，进一步提高政府效能。抓好行政法律法规的宣传教育，特别是贯彻实施好《行政许可法》，正确处理政府与企业、政府与市场、政府与社会、政府与公民的关系。自觉接受人民代表大会及其常委会的法律监督和工作监督。自觉接受人民政协的民主监督，搞好重大决策的政治协商，广泛听取社会各界意见。重视专家的建议和意见，提高决策民主化、科学化水平。加强与各民主党派、工商联、无党派爱国人士和人民团体的联系，充分发挥工会、共青团、妇联等群团组织的桥梁、纽带作用。

　　努力建设清廉型政府。坚持标本兼治，强化教育监督，深入推进党风廉政建设和反腐败斗争。坚持从严治政，加强执法监督和审计监督，坚决贯彻执行土地市场“招拍挂”、建设工程招投标和政府采购等制度，着力解决“公共权力部门化、部门利益化、利益个人化”问题，从源头上预防和遏制腐败。树立正确的政绩观，反对弄虚作假，严禁搞各种劳民伤财的“形象工程”、“政绩工程”。进一步从体制机制入手，坚决纠正部门和行业不正之风，防止和严厉查处以权谋私、权钱交易等违纪违法行为。牢记“两个务必”，始终保持谦虚谨慎，艰苦奋斗的作风，廉洁自律，勤政为民。

　　努力建设信用型政府。在政府及其工作人员中广泛开展诚信教育，使信用观念、信用意识、信用道德深入人心，增强政府行政的公信力。制定出台相应的规章和措施，加强对信用建设的政策支持和制度保障，加强对失信行为的惩治。把政府信用建设与依法行政结合起来，与深化行政审批制度改革结合起来，与推进政务公开结合起来，与政风评议结合起来，尽快在政府信用建设的运行机制、制度建设上取得实质性进展。通过建设信用政府，引导带动企业信用建设和个人信用建设，逐步把信用建设贯穿到社会各个阶层、各个行业，加快打造“信用赣州”步伐，在全社会形成“守信则荣、失信则耻”的信用环境。

　　各位代表，新的形势催人奋进，新的事业任重道远，新的使命光荣而又神圣。让我们紧密团结在以胡锦涛同志为总书记的党中央周围，高举邓小平理论伟大旗帜，深入贯彻“三个代表”重要思想，在中共赣州市委的领导下，全面实施“对接长珠闽、建设新赣州”发展战略，解放思想，振奋精神，同心同德，扎实苦干，为创造赣州更加美好的未来而努力奋斗！
